# Effect of manufacturing type and aging on the fit of metal frameworks: an in vitro Micro-CT study

**DOI:** 10.1007/s00784-025-06345-x

**Published:** 2025-04-25

**Authors:** Kübra Tokay Kızılırmak, Bahar Kavafoğlu Yılmaz, Kaan Orhan, Evşen Tamam

**Affiliations:** 1 Prosthodontics, Private Clinic, Ankara, Turkey; 2 Department of Dentomaxillofacial Radiology, Ankara University, Ankara, Turkey; 3 Department of Prosthodontics, Faculty of Dentistry, Gazi University, Ankara, Turkey

**Keywords:** Metal framework, CADCAM, SLM, Fit, Casting, Microcomputed tomography

## Abstract

**Statement of problem:**

It is very important to determine how the marginal and internal fit of the restoration just before cementation changes after the specified period and which technique preserves the fit of the metal framework, and it has not been discussed in the literature.

**Purpose:**

This in vitro study aims to evaluate the effect of conventional casting, CAD/CAM milling, and laser sintering manufacturing methods on the marginal and internal fit of Co-Cr metal frameworks and how the fit will be affected following thermomechanical aging using Micro-CT.

**Materials and methods:**

Digital impressions were taken from the prepared typodont and metal frameworks were designed with ExoCAD software. Co-Cr metal frameworks were manufactured with 3 production methods (*n* = 15). In order to reflect the clinical conditions, a ceramic firing simulation was performed on metal frameworks. Thermomechanical aging was applied to the samples, equivalent to 1 year of use. For statistical comparison, the differences between the manufacturing techniques ANOVA followed by Tukey and, for aging Wilcoxon Test were used (*α* = 0.05).

**Results:**

Compared to the conventional casting method, computer-aided methods showed a better fit. Thermomechanical aging increased misfit in all three production methods. At all determined points, before and after aging, the highest gaps were measured in the conventional casting method. In volumetric evaluations, both before and after aging, it was seen that the highest fit was in the laser sintering method, and the lowest fit was in the conventional casting method.

**Conclusions:**

Production technique and aging have an impact on the fit of metal substructures. Following aging, misfit increased more in computer aided manufacturing than conventional casting.

**Graphical abstract:**

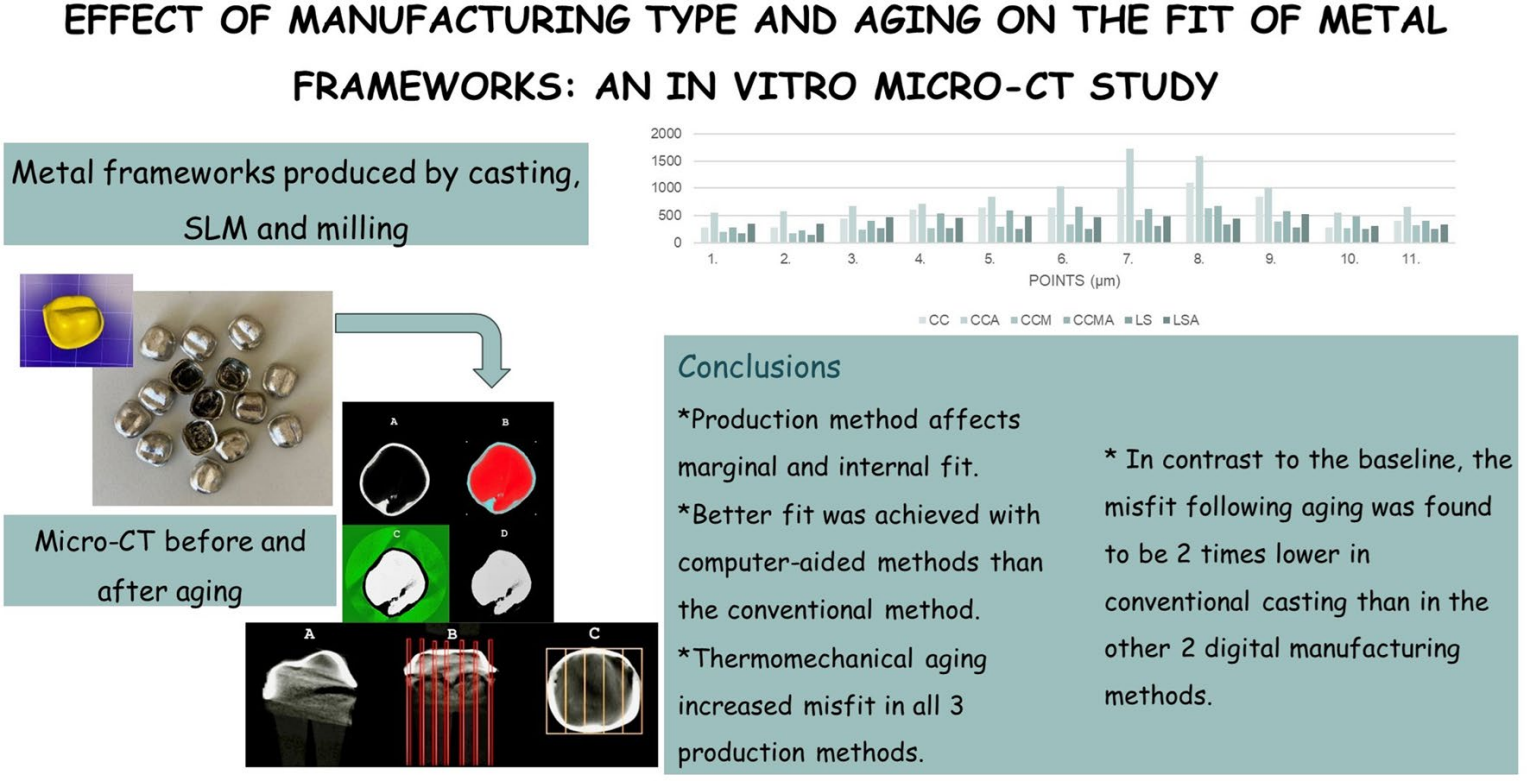

## Introductıon

Despite their advantages in aesthetic and biological aspects, metal based ceramic restorations (MCR) are still the most important alternatives to zirconia-supported restorations due to their biomechanical disadvantages, especially when used in long edentulous areas. MCR are frequently preferred in the clinic due to their economy, biocompatibility, aesthetic satisfaction, and resistance to high chewing forces, especially in posterior regions, implant-supported restorations, and long edentulous areas [1–3]. Restoration fit to the supporting structures is important in evaluating the long-term clinical success of fixed partial dentures. Even restorations that have a perfect fit at delivery may lose their fit over time due to oral conditions [4].

Preparation (i.e. marginal form, width of the preparation, taper value, the occlusal surface morphology of the prepared tooth), materials (i.e. type of burs used, impression materials and techniques, type of cement, fluidity of the cement and film thickness, the framework material itself, material thickness) and production (i.e. manufacturing method, the temperature changes during veneering and the difference between the thermal expansion coefficients, the calibration of the porcelain furnace and the number of porcelain firings), are only few factors affecting the marginal and internal fit of the MCRs [5–9]. In fact, there is a study in the literature showing that even the alloy composition affects marginal fit [10].

Conventional casting and computer-aided (additive and subtractive) manufacturing conditions in metal framework production also affect the marginal and internal fit [4, 11, 12]. In many studies comparing the production methods of metal-based restorations, it has been stated that metal frameworks produced by laser sintering have better compatibility than those produced by conventional casting and subtractive CAD/CAM methods [7, 11, 12]. While some studies report that metal frameworks produced with the milling system are better than those produced with 3D printers [8–10], there are also studies indicating that metal substructures produced by the conventional casting method are better than those produced by the other two methods in terms of fit [13, 14].

Although it has been frequently investigated how different production techniques affect the marginal and internal fit in MCRs, no study has been found in which all of these techniques were evaluated with the microcomputed tomography (µ-CT) method by simulating the ceramic firing process and chewing conditions. In addition, µ-CT examinations to be performed before and after aging are also very important in terms of determining how the marginal and internal fit of the restoration at the time of first delivery to the patient changes after the specified time and which technique preserves the marginal and internal fit of the metal substructure for a longer period.

This study aims to examine the differences between the internal and marginal fit of metal frameworks produced by conventional casting, CAD/CAM milling, which is a subtractive production method, and laser sintering, an additive manufacturing method, and how the fit will change in 1 year at micrometric and volumetric level with µ-CT. The null hypothesis of the study was that the manufacturing method would have no effect on the marginal and internal fit and that this fit would not change after 1 year of aging.

## Materıals and methods

**Fig. 1:**
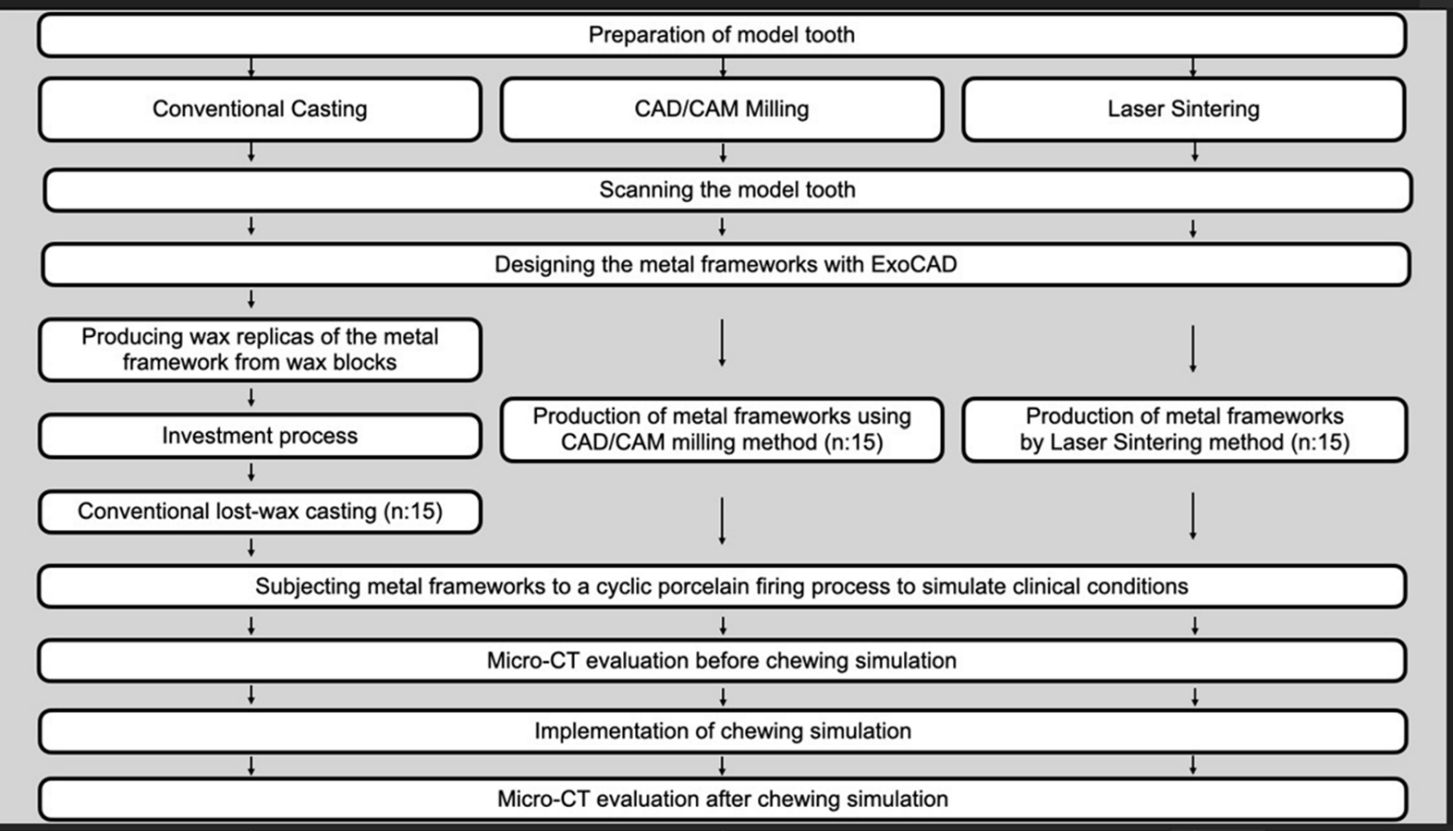
Workflow of the study

The 3 experimental groups (*n* = 15) determined by Power analysis (G* Power Software version 3.1.9.2) for effect size (d): 1.0, Power: 0.95, and α: 0.05, and flowchart are shown in Fig. 1.

The left lower 2nd molar typodont (Frasaco GmbH, Germany) was used as a model. Underwater cooling, a 2 mm occlusal, 1.5 mm axial, 1 mm supragingival width chamfer line, and 6° taper angle (total 12°) were prepared. The preparation was checked with a loop (orangedental GmbH, Biberach, Germany) and rounded with fine-grained burs, finishing burs, and rubbers (Frank Dental GMBH, Germany). The model was scanned with an optical scanner (3Shape R700 3D Scanner; 3Shape A/S Copenhagen, Denmark) to standardize the impression phase for manufacturing methods. The metal framework was designed using ExoCAD software (Exocad DentalCAD; Exocad GmbH, Darmstadt, Germany) with a thickness of 0.4 mm with a cement space of 25 μm.

**Fig. 2:**
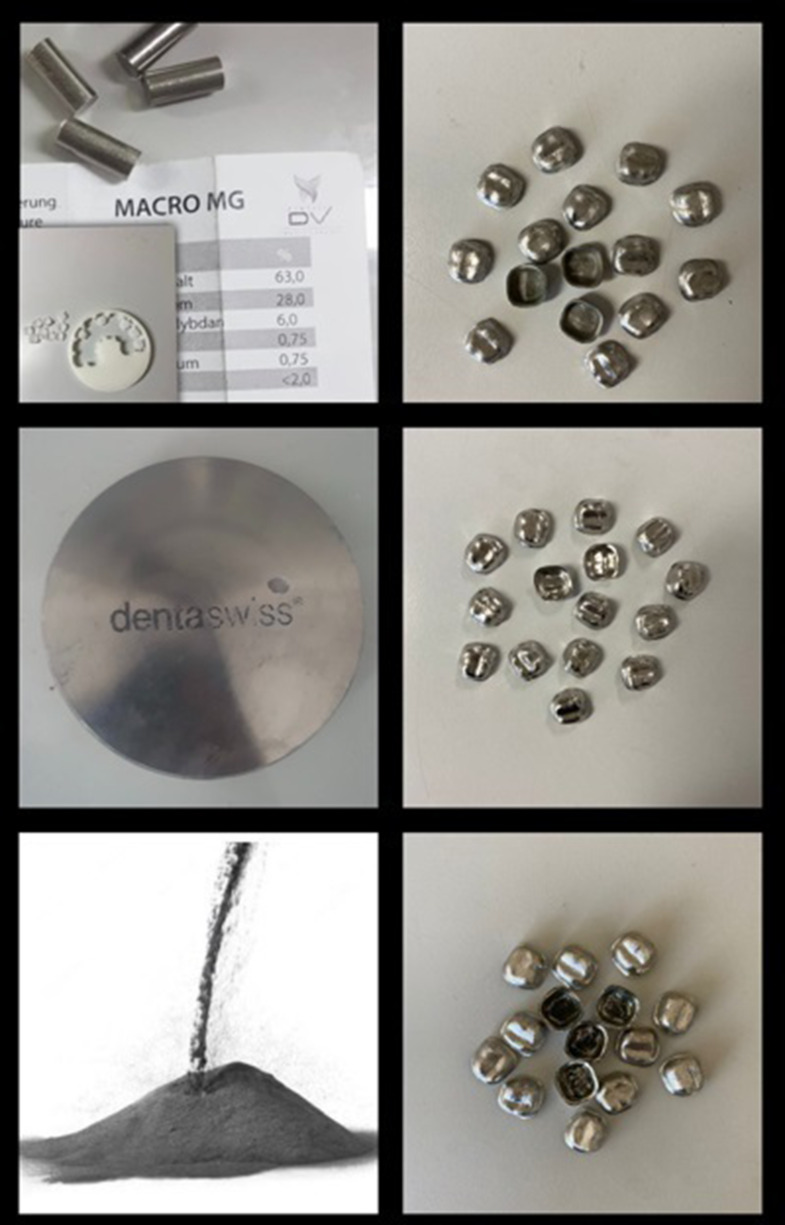
Materials and metal frameworks based on the 3 manufacturing methods

Materials and metal frameworks based on the 3 manufacturing methods are shown in Fig. 2. Metal frameworks were manufactured by conventional casting (CC), laser sintering (LS), and CAD/CAM milling (CCM) methods (*n* = 15) as follows:

CC Group: Wax replicas of the designed metal frameworks were produced on the milling device (Tizian Cut 5 smart; Schütz Dental GmbH, Germany). Finishing processes were carried out on metal frameworks obtained from Cr-Co alloy (Macro MG; DentVit Dental Group, Nuremberg, Germany) by conventional lost-wax casting.

CCM Group: Metal frameworks produced from prefabricated Cr-Co blocks (dentaswiss CoCr; Biodenta, Berneck, Switzerland), using a milling unit (Redon Hybrid; Redon Mühendislik, Türkiye) with a 5-axis rotary table, which can carry more than one burs at the same time, can choose the appropriate bur according to the material to be worked on, and the burs can be angled up to 30°. Following the production, the samples were carefully separated and finished.

LS Group: Metal frameworks were produced from Cr-Co alloy powders in a laser sintering printer (HBD-100D; Guandong Hanbang Laser Technologies Ltd, Shanghai, China). In the system with a fiber laser power of 160 W, a laser spot diameter of 60 μm, and a wavelength of 1060 nm, the oxygen in the forming chamber was reduced below 1000 ppm, and argon was used as a shielding gas. Following the production, the samples were allowed to reach room temperature. The metal connecting rods were separated and finished.

To reflect the clinical conditions, metal frameworks in all 3 groups were exposed to the porcelain firing cycle of one of the brands recommended by the manufacturer (Vita VMK 95^®^; Vident, California, USA) without stacking porcelain.

**Fig. 3:**
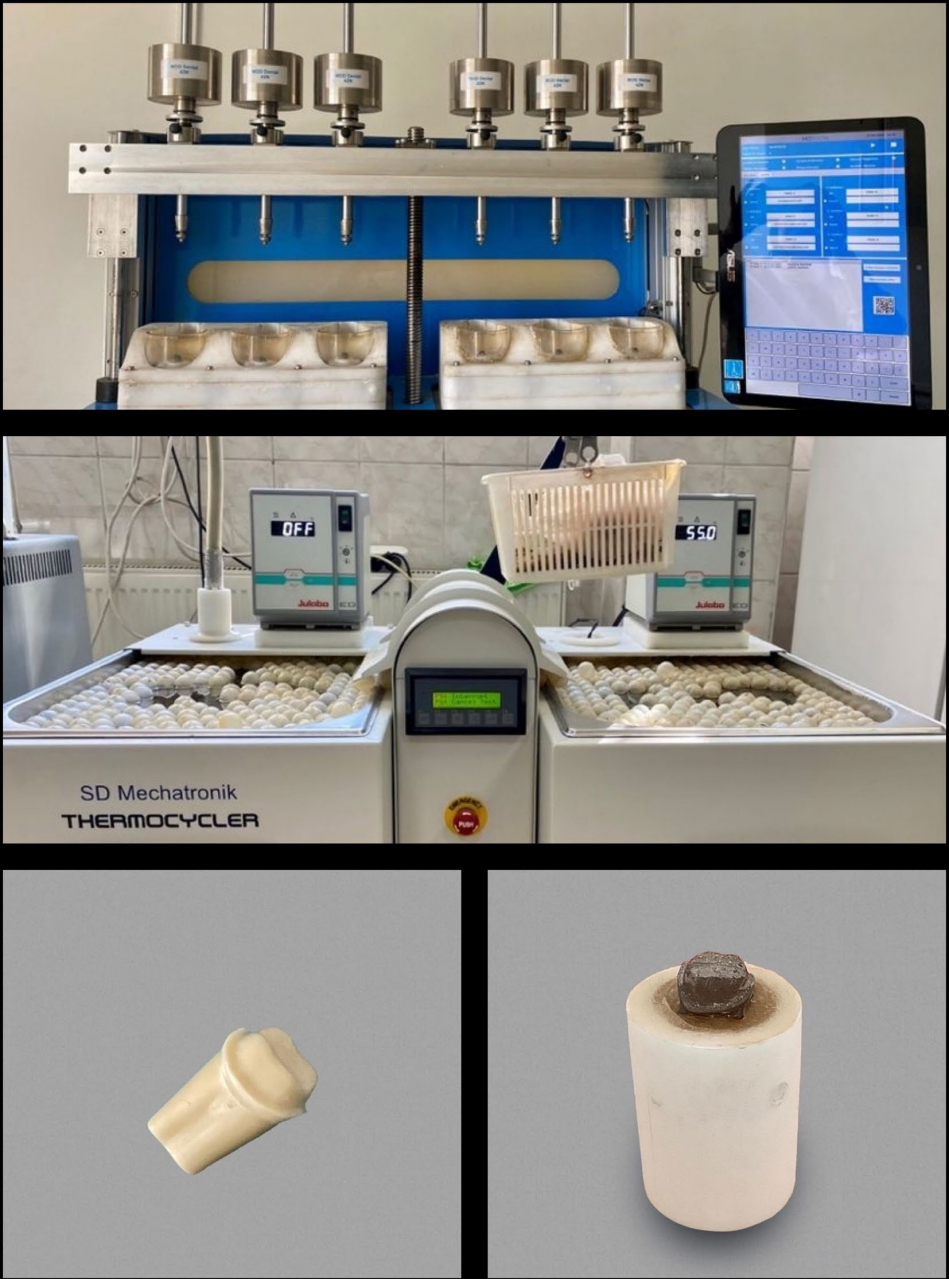
The rmomechanical aging set up and model tooth

Metal (Cr-Co) replicas of the working model were produced for use in the simulator. The replicas were fixed to the device in a vertical direction with acrylic resin (IMICRYL Dental; Konya, Türkiye) in cylindrical molds (Fig. 3). Metal frameworks were luted to replicas with temporary cement (Dycal; Dentsply Sirona, Konstanz, Germany). Artificial aging, in which mechanical and thermal processes were applied, was carried out in a chewing simulator (MOD; Esetron Smart Robotechnologies, Ankara, Türkiye) as 250 thousand cycles and 1000 thermal cycles, equivalent to 1-year usage [15]. Mechanical loading of 50 N was applied along the vertical axis with a 6 mm diameter steel ball at approximately 1.6 Hz. Thermal cycles were carried out to complete the cycle between 5 °C and 55 °C water in 1 min.

Before (following porcelain firing) and after aging, a high-resolution, desktop micro-CT system (Skyscan 1275, Bruker, Kontich, Belgium) with ring artifacts correction was used at 100 kVp, 100-mA beam current, 0.5-mm Al/Cu filter, 8.1 μm pixel size, and rotation at 0.5 step. Each sample was rotated 360° within a 10-minute integration time. The NRecon software (version 1.6.10.4, SkyScan, Bruker, Kontich, Belgium), DataViewer software (version 1.5.6.2, SkyScan, Bruker, Kontich, Belgium), and CtAn (version 1.17.7.2, SkyScan) were used for the visualization and quantitative measurements of the samples, which used the modified algorithm described by Feldkamp et al. [16] to obtain axial, two- dimensional, 1000 × 1000-pixel images. After computerized reconstruction, the images were superimposed using DataViewer. The first scanning of the teeth was used as a reference point and superimposed after scans with crown restorations (target); this generated a volume of subtracted images. This image represented the entire area and volume of the gap between the crown and teeth. Following, the CTAn was used for the 3D volumetric visualization, analysis, and volume measurement (mm^3^).

**Fig. 4:**
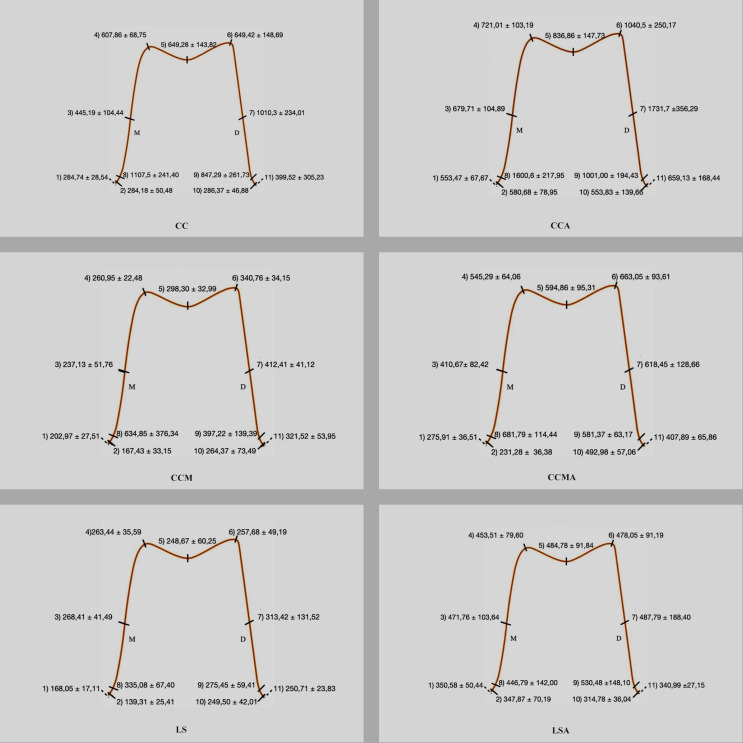
2D (micrometric) analysis of the gap between the metal substructure and the supporting tooth at selected points for 3 different production methods, before and after the chewing simulation

Besides, DataViewer was used for 2D micrometric analysis to measure the distance between the metal substructure and the support tooth (µm). The 11 measurement points where internal and marginal fit could be interpreted were analyzed (Fig. 4). Measurements were repeated at each point 3 times, and the means were noted. In total, 255 measurements were done for each crown.

Statistical evaluations of the measurements obtained from all samples were made using IBM SPSS Statistics 22 (IBM SPSS). Marginal and internal fit values obtained after porcelain firing and aging from metal substructures produced with different manufacturing techniques were compared statistically. The Shapiro-Wilk normality test was used to determine the distribution of the data. One-way ANOVA for normally distributed values in the comparison of the effect of production methods; Tukey tests were used when the variances were homogeneous, and Tamhane tests were used if the variances were not homogeneous. Kruskal Wallis test was used for values that did not show normal distribution, and Dunn’s test was used to determine from which group the difference originated (α = 0.05). In the comparison of the effect of aging, the Paired T-test was used for normally distributed values, and the Wilcoxon Test was used for non-normally distributed values (α = 0.05).

## Results

**Fig. 5:**
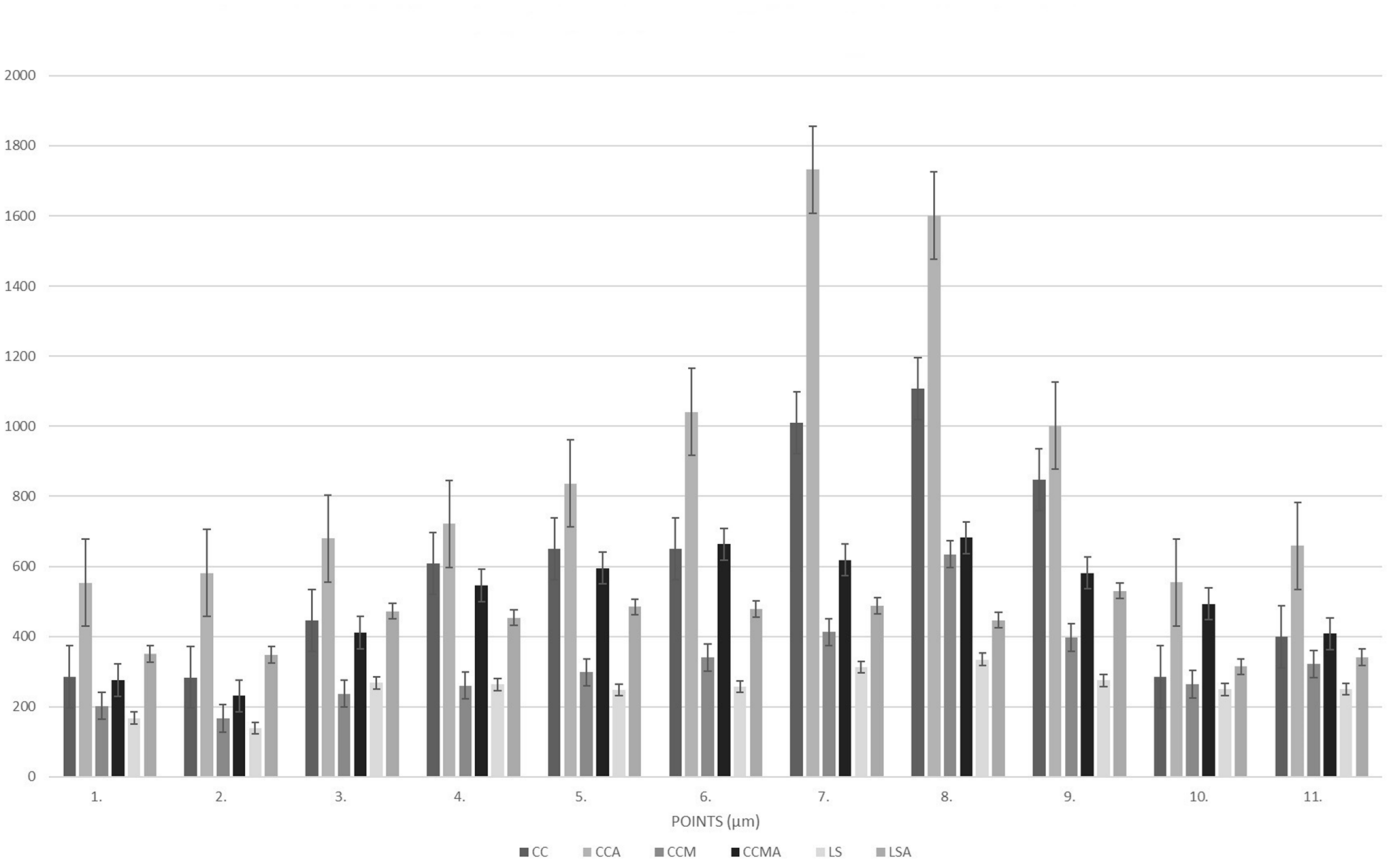
Graphical view of 2D (micrometric) analysis of the gap, before and after the chewing simulation

The means obtained from 2D and 3D analyses are shown in Table 1; Figs. 4 and 5.

**Table 1:** The 3D (volumetric) Micro-CT results, show the volume of the space between the metal framework and the supporting tooth

	Production Method	Mean (mm^3^)	Min (mm^3^)	Max (mm^3^)	Between Groups	Difference	*p*
Before Chewing Simulation	CC^A^	5,16 (± 0,50)	4,143	5,944	CC-CCM*	2,04 (± 0,53)	< 0.001
	CCM^B^	3,12 (± 0,26)	2,699	3,564	CC-LS*	2,25 (± 0,47)	< 0.001
	LS^C^	2,91 (± 0,32)	2,315	3,374	CCM-LS	-0,21 (± 0,41)	= 0.274
After Chewing Simulation	CC^a^	11,90 (± 0,65)	10,641	12,955	CC-CCM*	6,27 (± 0,99)	< 0.001
	CCM^b^	5,63 (± 0,56)	4,584	6,597	CC-LS*	7,29 (± 0,91)	< 0.001
	LS^c^	4,62 (± 0,58)	3,663	5,837	CCM-LS*	1,01 (± 0,72)	< 0.001

^A, *^Superscripts indicate statistical significance within the same column and stars within the same row

**Fig. 6:**
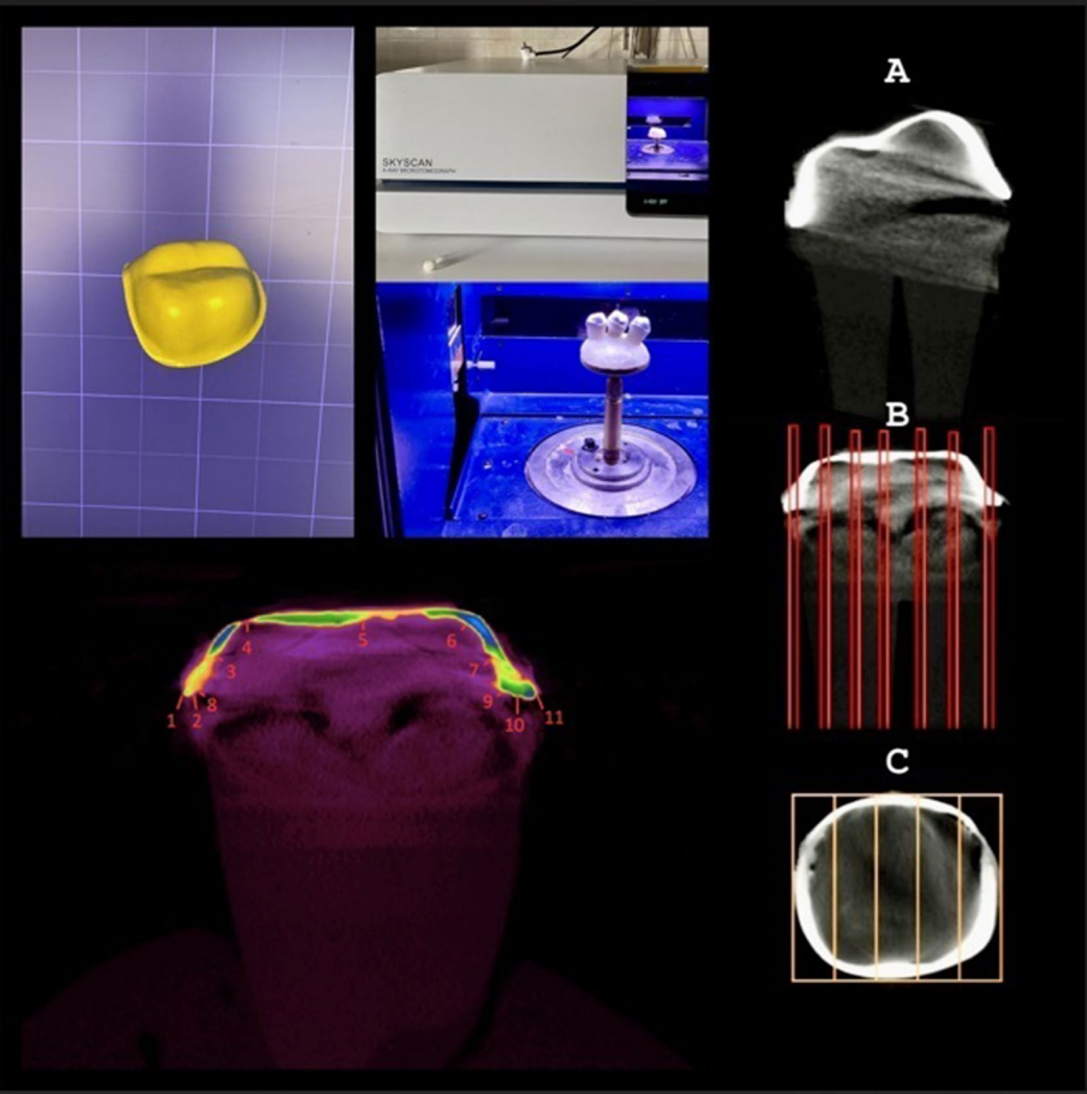
Representative of 2D and 3D micro CT measurements

According to the 3D analyses, in which the volume of the space between the metal framework and the supporting tooth is calculated (Fig. 6), both before and after the chewing simulation, the production methods are listed in decreasing order as CC > CCM > LS. For the CC group, the higher misfit at the beginning (5.63 ± 0.5 mm³) and following the chewing simulation symbolizing 1-year use (11.9 ± 0.65 mm³) was statistically significant compared to the other 2 methods. Although not statistically significant, the initial discrepancy calculated in CCM (3.12 ± 0.26 mm³) was slightly higher than in LS (2.91 ± 0.32 mm³). After aging, the difference between these 2 methods (1.01 ± 0.72 mm³) was statistically significant.

According to 2D analyses that measure the distance between the metal substructure and the supporting tooth, both before and after aging, the highest discrepancy was detected with CC, and for the 11 selected points, almost all of the differences were statistically significant (Figs. 4 and 5).

In all manufacturing methods tested, the aging procedure increased the volume of the space (3D) between the metal framework and the abutment tooth (Table 1). The aging process caused an increase of 6.75 ± 0.60 mm³ in CC, 2.51 ± 0.41 mm³ in CCM, and 1.71 ± 0.75 mm³ in LS and for all 3 methods; the differences were found to be statistically significant (Table 1). When looking at the micrometric (2D) effects of the aging procedure, it was seen that for each of the 11 points selected in all 3 production methods, the discrepancy increased with aging, and almost all of the differences were statistically significant (Figs. 4 and 5).

## Discussion

One of the most important features that fixed partial dentures must have to be long-lasting is that the restoration does not lose harmony with the supporting tissues. Distances exceeding the optimum value (> 120 μm) create the necessary conditions for biological complications [17]. In casting processes or during the cooling of the cast metal, the cement gap may differ from the planned due to thermal shrinkage, and the marginal gap may be above acceptable limits [18]. Therefore, marginal discrepancies are often inevitable in the CC process due to many clinical and laboratory procedures. Supporting this information, in our study, the marginal gap measured at the beginning (before aging) was higher than planned at the design stage for all tested production methods (Table 1; Figs. 4 and 5).

MCRs gain aesthetic properties by firing ceramics in layers at high temperatures on a metal substructure. These firing degrees to which substructures are exposed affect the marginal and internal fit. Although ceramic systems that are thermally compatible with the substructure are selected, it has been stated in the literature that even heat treatment alone can affect marginal and internal harmony [19]. Exposure to high temperatures negatively affects the fit of MCRs by causing deterioration in the metallurgical structure of the alloy [20–22]. In our study, it can be claimed that Micro-CT measurements should be made before firing to see the effect of heat treatment on fit. Since MCRs are delivered to the patient in the clinic after the ceramic is fired, making the initial measurement on fired samples is not a disadvantage for this study. One of the goals of our study was to determine how the fit of an MCR will change over time from the moment of delivery.

To simulate clinical conditions as much as possible, a ceramic firing cycle was applied to metal substructures in our study. In this process, which was carried out without the application of ceramic, the temperatures of one of the ceramic systems (Vita VMK 95^®^) recommended for all 3 techniques were applied for the prescribed periods. Sasany and Yılmaz [23] showed a decrease in marginal fit after veneering. Önöral et al. [8] also state that the marginal fit of three-unit MCR substructures produced with 3 different CAD/CAM and CC methods decreases following repeated firings. In a study examining the effect of repeated firing on the marginal fit of metal substructures produced by conventional casting and computer-aided methods [24], it was found that firing had no impact on the marginal spacing, but a better marginal fit could be achieved with computer-aided production. This result is attributed to the fact that the conventional casting method is a complex and sensitive technique that requires more steps than others. In our study, the designed marginal gap increased remarkably even in the CC group, where the wax sample was prepared with computer aid, and in the CCM and LS groups, where the wax sample was not used (Figs. 4 and 5). The marginal gap, which was set at 25 μm at the design stage and increased by 11 to 44 times in the CC group, 7 to 25 times in the CCM group, and 6 to 13 times in the LS group, can be attributed to the heat treatment applied to the samples rather than laboratory processes (Figs. 4 and 5). Compared to the other 2 groups, the highest discrepancy obtained in the CC group may indicate possible deformation of the wax in the following processes, even if it is prepared untouched, and additional distortions in the cooling phase with the high temperatures reached during the casting processes. The least mismatch (highest fit) among metal substructures produced by different methods and exposed to temperature under equal conditions was seen in the LS group. It should be kept in mind that if the study is carried out by stacking ceramics, the results may change due to the high specific heat capacity and low thermal conductivity of ceramics.

A more interesting result was found when baseline (symbolizes the moment of delivery to the patient) and post-aging (symbolizes 1 year of use) were compared. Although the values in the CC group increased in both the marginal and internal regions, it is noteworthy that the aging process caused an almost 2-fold increase in disharmony in both the CCM and LS groups (Table 1; Figs. 4 and 5).

Tekin and Hayran [25] examined the marginal fit of monolithic zirconia and Ni-Cr MCR crowns with different thicknesses and found that the marginal gap was statistically significantly higher in MCR samples compared to all other groups. The ceramic firing process is thought to have a negative effect on the fit in our study. However, in our study, the firing process was carried out as a simulation that only reflected temperatures without using ceramic, which may have resulted in not being able to benefit from the metal’s compression feature of ceramic due to thermal expansion and resulting in wider gaps. The results may vary when ceramic is fired in layers on a metal substructure.

Both 2D and 3D measurements can be made with Micro-CT. It is possible to make measurements on 2D sections taken at certain resolutions and numbers and to make volumetric evaluations with 3D images obtained by combining and restructuring these sections. It is possible to simultaneously make measurements in a single axis (X or Y) direction with 2D sections and in 3 axes (X, Y, and Z) direction from 3D images [26]. Micro-CT allows 2D sections to be adjusted to exactly the same coordinates in each sample [21]. In our study, 2D sections were obtained by setting the same coordinates in each sample. Distance measurements, expressed as points 1–11, were made from the sections that can be measured in a single-axis direction (Figs. 4 and 5). It should be noted that if the coordinates are changed or the samples are rotated, the measured values will change. At this point, it may be questioned how orientation and matching are performed in distance measurements in 2D analyses. In our study, this issue was carefully managed by using the DataViewer software to superimpose all images systematically. The reference scan of the tooth without the crown was utilized as the baseline, ensuring that subsequent scans with the crown were aligned precisely using defined anatomical landmarks. This alignment minimized variability due to orientation. Additionally, to ensure reproducibility and reduce operator bias, measurements at each of the 11 points were repeated three times, and the mean values were used for analysis. These measures ensured consistent matching across all samples and significantly minimized the impact of orientation variability on the 2D micrometric distance measurements. Volume measurements were carried out from 3D images, which can be measured in all 3 axes simultaneously (Table 1). No matter where the sample is placed, volumetric values are constant since measurements can simultaneously be made from all 3 directions. Our study evaluated volume measurements expressing total misfit separately regarding 3 production methods and aging factors.

In our study, 2D measurements were obtained by setting consistent coordinates for all samples using DataViewer software. However, as acknowledged, 2D measurements are inherently limited to single-axis (X or Y) evaluations and are sensitive to minor discrepancies in sample orientation or alignment. While we addressed this by maintaining consistent coordinates and repeating measurements, some degree of variability cannot be entirely eliminated in 2D analysis.Conversely, 3D measurements provide a more robust alternative by evaluating the gap in all three spatial dimensions (X, Y, and Z) simultaneously. This approach eliminates the dependence on precise orientation or sectioning, as the volumetric data inherently accounts for variations across all axes. Furthermore, 3D measurements are less affected by minor positional changes during scanning, making them more reliable for assessing the total misfit.In summary, while 2D measurements offer detailed insights into specific locations, 3D measurements provide a comprehensive evaluation of overall fit and are less prone to errors caused by orientation or matching inconsistencies. This highlights the necessity of interpreting both analyses together, as we have done in this study, to gain a holistic understanding of the fit characteristics of dental frameworks.

In our study, the initial gap volume measured for the CC method increased by 6.75 ± 0.60 mm^3^ following aging. This volumetric change due to aging was measured as an increase of 2.51 ± 0.41 mm^3^ for the CC method and 1.71 ± 0.75 mm^3^ for the LS method, and the differences are statistically significant for all 3 production techniques. For all 3 production methods, the decrease in fit following aging raised approximately 2-fold (Table 1). In the study to evaluate the effect of the production method and thermomechanical aging on marginal fit, the initial marginal fit values were similar for all groups, while the aging process affected the fit to varying degrees [23]. Although different materials and production methods were used, thermomechanical aging increased the marginal mismatch in all 3 groups in our study.

It has been reported that in the marginal and internal harmony of 3-unit Co-Cr bridges produced by CC, CCM, and DMLS (direct melting laser sintering) methods, the best fit was obtained with the DMLS method, and the manufacturing method creates statistically significant differences in the fit [27]. Nesse et al. [28] evaluated the marginal and internal fit of Co-Cr metal substructures produced by CC, SLM, and CCM methods with silicon replica and light microscopy. In the study where it was found that the SLM method was the weakest and the milling technique was the best, this result was associated with the fact that the metal beads that may have remained on the inner surfaces of the metal substructures produced with the SLM technique may have affected the passive fit. In the study evaluating the marginal and internal fit of implant-supported crowns produced with four different manufacturing techniques (CC, induction casting, plasma casting, and CCM), it was reported that the best results belonged to the CCM manufacturing technique [29]. In our study, it was found that manufacturing methods affected total fit. The gap volume before chewing simulation was measured as 5.16 ± 0.50 mm^3^ for CC, 3.12 ± 0.26 mm^3^ for CCM, and 2.91 ± 0.32 mm^3^ for LS. Although not statistically significant, the LS group fits higher than the CCM group. The fit observed in the CC group was statistically lower than the other 2 manufacturing methods. After the chewing simulation, the differences between the 3 groups listed in the same way in terms of gap volume (CC > CCM > LS) are statistically significant (Table 1).

The data obtained in our study showed that manufacturing methods impact marginal and internal fit. It has been observed that, as expected, computer-aided methods can achieve more precise adaptation than the conventional method. The LS method, sintered by accumulating powder in layers, was better than CCM in terms of fit. It was observed that the loss of fit increased following aging, corresponding to 1 year of use for all 3 methods. When considered proportionally, the loss of fit at the end of 1 year with the conventional casting method was less than with computer-aided systems. The partial differences between the distance measurements made only according to the selected coordinates in the 2D analyses and the gap volume measurements made independently of the location in the 3D analyses showed that both analyses should be interpreted together. If a single analysis is to be performed, volume analysis is considered more sensitive. This study has some limitations such as using a single type of alloy, ceramic firing as a simulation, and the duration of the aging protocol. Within the scope of this study protocol, studies evaluating different alloy types, different analysis methods, real firing of ceramic layers, and analysis at certain intervals by making longer-term aging protocols to monitor how the harmony changes over time are carried out will provide comprehensive information.

## Conclusions

Based on this study, the following conclusions can be listed:

1. Manufacturing method affects marginal and internal fit.
2. Better fit was achieved with computer-aided methods than the conventional method.
3. Thermomechanical aging increased misfit in all 3 manufacturing methods.
4. In contrast to the baseline, the misfit following aging was found to be 2 times lower in conventional casting than in the other 2 digital manufacturing methods.

## Data Availability

No datasets were generated or analysed during the current study.
